# Recent Progress in Wearable Near-Sensor and In-Sensor Intelligent Perception Systems

**DOI:** 10.3390/s24072180

**Published:** 2024-03-28

**Authors:** Jialin Liu, Yitao Wang, Yiwei Liu, Yuanzhao Wu, Baoru Bian, Jie Shang, Runwei Li

**Affiliations:** 1 CAS Key Laboratory of Magnetic Materials and Devices, Zhejiang Province Key Laboratory of Magnetic Materials and Application Technology, Ningbo Institute of Materials Technology and Engineering, China Academy of Sciences, Ningbo 315201, China; liujialin@nimte.ac.cn (J.L.); wangyitao@nimte.ac.cn (Y.W.); liuyw@nimte.ac.cn (Y.L.); wuyz@nimte.ac.cn (Y.W.); bianbr@nimte.ac.cn (B.B.); 2 College of Materials Science and Opto-Electronic Technology, University of China Academy of Sciences, Beijing 100049, China; 3 Materials and Optoelectronics Research Center, University of Chinese Academy of Sciences, Beijing 100049, China

**Keywords:** functional sensor, near/in-sensor computing, flexible electronics, neuromorphic perception system

## Abstract

As the Internet of Things (IoT) becomes more widespread, wearable smart systems will begin to be used in a variety of applications in people’s daily lives, not only requiring the devices to have excellent flexibility and biocompatibility, but also taking into account redundant data and communication delays due to the use of a large number of sensors. Fortunately, the emerging paradigms of near-sensor and in-sensor computing, together with the proposal of flexible neuromorphic devices, provides a viable solution for the application of intelligent low-power wearable devices. Therefore, wearable smart systems based on new computing paradigms are of great research value. This review discusses the research status of a flexible five-sense sensing system based on near-sensor and in-sensor architectures, considering material design, structural design and circuit design. Furthermore, we summarize challenging problems that need to be solved and provide an outlook on the potential applications of intelligent wearable devices.

## 1. Introduction

With the rapid development of the Internet of Things (IoT), health monitoring devices such as armbands and glasses are becoming more intelligent and accurate. With the pressing need for high accuracy and real-time detection in medical applications, wearable and implantable devices are rapidly developing due to their advantages in directly collecting physiological information [1,2,3,4,5,6,7,8,9,10,11]. Through direct contact with the skin or even organs, wearable sensors can reduce the effect of interference and environmental noise compared to other general sensors. For wearable devices used in health monitoring, some characteristics should be fulfilled, including the following: (1) The device should have good stretching and bending properties to adapt to a range of human daily routines. (2) The device should have good biocompatibility to avoid skin irritation. (3) The device should be lightweight and comfortable to ensure comfort [12,13,14,15,16,17]. Recent research has shown that health monitoring devices have achieved high performance by integrating high-precision wearable sensors and computers [18,19,20]. However, redundant data being output from sensors is inevitable, and this causes a high transmission latency and power consumption to occur between sensors and computing units. To alleviate the shortcomings outlined above, a new computing paradigm proposes integrating some computing units into the sensing unit. Architecture differences and the classification of near/in-sensor computing can be distinguished by the degree of integration [21]. Zhou et al. referred to this part of the computing unit as the front-end processing unit. The front-end processing unit can filter the redundant data and extract the effective data output from the sensors [22,23,24]. In addition, some researchers have proposed a variety of smart sensors that can perform summation operations by designing materials and structures, which are called in-sensor computing system [25,26,27]. As a new generation of smart sensor units, near/in-sensor computing systems have great potential for use in various applications, such as medical equipment, bionic robots and electronic skin.

Devices or circuits with information processing capabilities are required for the front-end processing unit. Memristors and synaptic transistors have emerged as the best choice for front-end processing units in near-sensor computing systems due to their ability to simulate the synapses of neurons [28,29]. If the postsynaptic current generated from redundant sensor data activated in near/in-sensor computing systems is not large enough, this means that it can be filtered out [30,31,32]. Until now, researchers have conducted systematic work on near/in-sensor computing systems, demonstrating the feasibility of tactile, visual, auditory and olfactory systems which use near/in-sensor computing [33,34,35,36,37,38]. However, hard bending or stretching is a general disadvantage in most of the recently proposed near/in-sensor computing systems due to their rigid electronic elements. The equipment used in health monitoring applications should be deformable and biocompatible. Therefore, the challenge of developing materials and electronic components which fulfill both good flexibility and biocompatibility still remains in this field. Additionally, research on memristors and synaptic transistors has primarily focused on rigid metal oxide materials due to the specific mechanisms of current neuromorphic devices. Recently, researchers have discovered the potential of near/in-sensor computing systems in edge computing and progress has been made in the flexibility of all components [39,40]. Due to their different working mechanisms, flexible sensors in system can be identified as piezoresistive, capacitive, piezoelectric or triboelectric sensors [41,42,43,44]. Filling a flexible medium layer with functional filler and depositing a film of functional materials on a flexible substrate are the two main methods of flexible material preparation. Carbon-based materials such as graphene, fullerene, C-nanotubes and carbon quantum dots have been more popular in recent research due to their excellent compatibility with elastic materials [45,46,47,48]. Additionally, metal powders or nanowires such as Cu, Au, Ag and Pt can be selected as functional fillers to confer electrical, magnetic or thermal properties to the functional medium layer. For instance, insulating materials can acquire conductive properties by being coated or mixed with conductive materials [49,50,51]. Notably, the combination of high-performance elastic materials and functional materials has emerged as a novel approach for fabricating high-performance flexible devices. Reducing the thickness of rigid functional materials such as ITO, mica and metal conductors is a method used to obtain stretching and bending properties. For example, metal oxide epitaxial thin films grown on flexible substrates also exhibit good flexibility [52,53,54]. In order to alleviate the concentration of stress, the thickness of rigid materials can be thinned, which can significantly reduce defects. In addition to the material used, designing special structures can also improve the performance of flexible sensors. Flexible sensors typically present an inferior mechanical performance and stability compared to rigid sensors; however, the introduction of microstructures such as pyramids, cylinders, half-spheres and wrinkles significantly enhances the resilience, stability and sensing capabilities of flexible sensors, which can be helpful for the development of high-performance flexible sensors [55,56,57]. Currently, the integration of material design and structural design is the most prevailing approach for designing flexible sensors in near/in-sensor systems.

Neuromorphic devices are an essential component of near/in-sensor systems due to their information-filtering ability and processing capabilities. Like flexible sensors, these devices require good flexibility and stability. Two completely different technical routes can be adopted for the fabrication of flexible memristors and synaptic transistors for use in the system. Flexible memristors are typically prepared by depositing electrodes and functional metal oxide films such as nickel oxide, hafnium oxide and titanium oxide directly onto a flexible substrate [58,59,60]. Nevertheless, for synaptic transistors, researchers are exploring novel flexible functional materials, such as organic semi-conductors, graphene composites and bio-based materials, to replace the semi-conductor layer and substrate in conventional transistor structures [61,62,63]. Furthermore, there are intrinsic differences between memristors and synaptic transistors, influencing researchers’ choices. Memristors can achieve higher integration densities due to their 2-terminal with a simple layered structure, while synaptic transistors offer a better design in terms of flexibility and stability due to their wide range of material selections and gate control mechanisms [64]. Flexible memristors and synaptic transistors have their own advantages in different applications. Research on near/in-sensor computing systems is still in the exploratory stage. 

**Figure 1:**
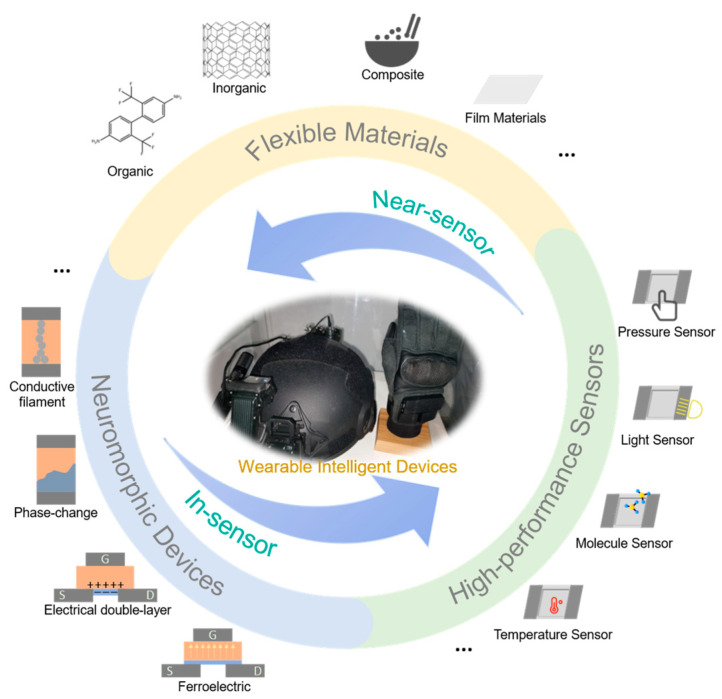
Design thinking diagram for wearable near/in-sensor intelligent systems.

In conclusion, constructing a wearable near/in-sensor perception system is a complex task due to the incorporation of flexible materials, neuromorphic devices and high-performance sensors (Figure 1). Currently, the design of wearable near/in-sensor perception systems is mainly based on material selection and structural design because the tensile and flexural properties of the material can directly determine the flexibility and stability of the system, and the appropriate structural design can help to improve the performance of the components, and, thereby, the whole system. This review focuses on recent advancements in flexible sensors and neuromorphic devices’ utility of near/in-sensor perception systems. Starting with the basic structure and working mechanism, we provide an overview of material design, structure design and near/in-sensor computing systems application. We summarize the main challenges in this sphere and provide an outlook on the application prospects of near/in-sensor computing systems. See Table 1.

## 2. Near-Sensor and In-Sensor Computing System

In 2020, the research on near/in-sensor computing systems by Zhou et al. indicated that these systems are primarily designed to solve the problem of large amounts of redundant data being exchanged between sensory and computing units [21]. Compared to the conventional sensor–Analog-to-Digital convertor (ADC)–memory–backend architecture, the ADC and memory capabilities of sensory and computing units are replaced by neuromorphic devices in emerging near/in-sensor computing architectures. Neuromorphic devices such as memristors and synaptic transistors can be easily integrated into sensory units due to their simple structure. Additionally, these devices have an extremely low power consumption as they operate in a micro-current [23,65,66,67]. In addition to filtering, the ability neuromorphic devices to perform some information processing exists to some extent. In other words, neuromorphic devices can simulate synapse models to process information input. They have synaptic plasticity and nonvolatile characteristics which allow them to process specific voltage signals as input and store output in resistance form [68,69,70].

The relationship between near-sensor computing and in-sensor computing is progressive. A near-sensor computing system can filter redundant data at the sensing terminal by integrated the partial computing units near to the sensing units. This design significantly reduces the computing stress on computing units and the transmission consumption between sensing units and computing units. The necessity of a multi-functional sensing unit in in-sensor computing systems is to fulfil both ambient signals inputting and perform simple computing. This design eliminates the consumption caused by redundant data transmission. Neuromorphic devices can replace the ADC unit in near/in-sensor computing systems to decrease power consumption further by processing analog signals directly. Numerous experiments have demonstrated that integrating the neuromorphic computing paradigm with near/in-sensor architecture can significantly decrease power consumption by 1~2 orders of magnitude [71,72,73]. As a result, a neuromorphic device with a small size has better prospects for use in the self-powered devices in wearable technology [74].

## 3. Flexible Near-Sensor Artificial System

The sense organ system is a crucial part of the human body, acting as a primary interface between the body and the external environment. The human body’s sense organ system can be broadly categorized into five types based on their working mechanisms: the tactile system (pressure and strain responsive), the temperature system (somatosensory), the visual system (light responsive), the auditory system (acoustic responsive) and the olfactory and gustatory system (molecular responsive) [75,76]. In comparison to artificial intelligence, the human nervous system is still more energy efficiency [77]. Near/in sensor systems, inspired by human sense organ system, have significant potential in the IoT. To create wearable near/in-sensor systems which are compatible with diverse signal inputs, flexible sensor fabrication often adopts material and structural design approaches. Diverse inputs have their counterpart outputs; therefore, matching sensors and front-end processing units can be a challenge in near/in-sensor computing systems design.

### 3.1. Flexible Near-Sensor Tactile System

**Figure 2:**
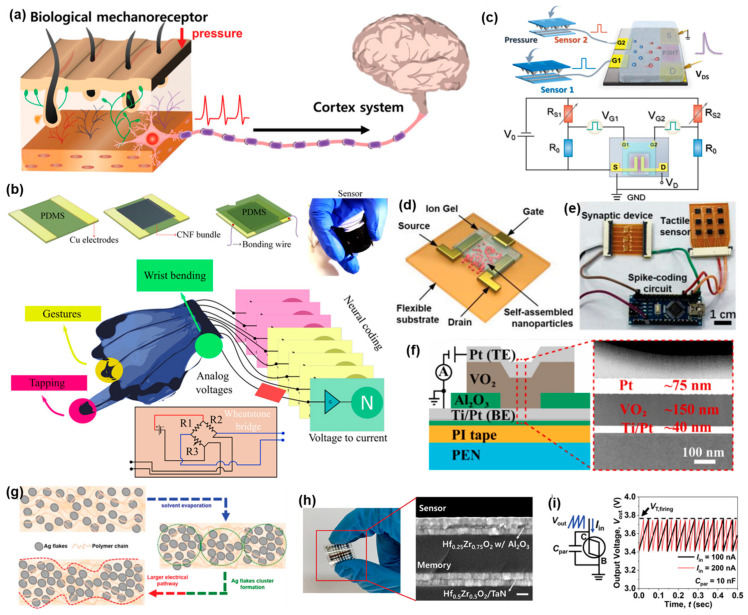
Near-sensor tactile system. (**a**) Neural pathway of tactile system in human beings. Reprinted with permission from Ref. [99]; Copyright 2022 John Wiley and Sons. (**b**) Schematic image of intelligent glove with Wheatstone bridge oscillators as front-end processing units. Reprinted with permission from Ref. [90]; Copyright 2022 American Chemical Society. (**c**) Flexible sensors and ion-gate transistor with signal matching circuit. Reprinted with permission from Ref. [91]; Copyright 2023 John Wiley and Sons. (**d**) Structural diagram of ion-gate transistor. (**e**) Flexible sensors and transistor array with spike-coding circuit. Reprinted with permission from Ref. [92]; Copyright 2022 John Wiley and Sons. (**f**) Structural diagram of memristor as front-end processing unit. Reprinted with permission from Ref. [93]; Copyright 2022 Elsevier. (**g**) Structural diagram of a flexible sensor used in near-sensor system. Reprinted with permission from Ref. [94]; Copyright 2021 John Wiley and Sons. (**h**) Diagram of flexible near-sensor system prepared using inorganic materials. Scale bar: 100 nm. Reprinted with permission from Ref. [98]; Copyright 2024 John Wiley and Sons. (**i**) Self-powered near-sensor system and its synaptic characteristics. Reprinted with permission from Ref. [99]; Copyright 2022 John Wiley and Sons.

The tactile system is a complex sensor organ system consisting of various receptors such as Pacinian corpuscles, Herbst corpuscles and Merkel disks [78] (Figure 2a). Recent flexible near-sensor system research has been met with the difficulty of integrating individual sensors with these complicated functions. However, research on near-sensor systems with single tactile functions has gradually improved [79]. As a pressure perception system, the four basic pressure perception mechanisms which exist for near-sensor system construction have their own advantages. The piezoresistive mechanism has a simple principle; fundamentally, the resistance of the piezoresistive sensors will be changed along with pressure through a specific function, which is generally negative relevant. Because of their simple structure and wide selection of materials, the preparation and integration of piezoresistive sensor are easy in a large-scale system [80]. The sensor function layer (SFL) is the material between two electrodes which ensures that the piezoresistive sensors are working properly. A flexible SFL with conductivity can be made of some conductive materials such as graphene, carbon nanotubes or carbon nanofiber [81,82,83] and combined with flexible organic polymer materials like polydimethylsiloxane (PDMS), thermoplastic polyurethane (TPU) or resin [84,85,86]. What is more, optimizing the piezoresistive sensors’ performance requires a microstructure design [87,88,89]. Similarly, the design of flexible front-end processing units is also important for implementing near-sensor perception systems. Both neuromorphic devices and logic circuits can filter redundant data from the sensing terminal. Sengupta et al. [90] designed a flexible and stretchable electro-spun carbon nanofiber (CNF) sensor for an intelligent perception system. This sensor has a simple sandwich structure with a PDMS layer by bonding a CNF bundle to copper tape electrodes on two edges and encapsulating them. It will output a voltage of approximately 0–1.2 V in response to a bending angle of 0–90°. This flexible sensor array with a crisscross structure can also be implemented for gesture detection and recognition. A Wheatstone bridge oscillator can generate several numbers of spikes with the voltage output from the sensor (Figure 2b). This mechanism allows pressure and bending signals to be transferred as spikes, and the capacity of the signal will accumulate to reach a threshold and trigger another signal to the back-end units (e.g., Artificial Neural Network). This work testifies to the various gestures involved and allows us to obtain the number of spikes in their curves synchronously. Different curve shapes and diverse gestures are the conditions used for classification. A near-sensor tactile system designed by Liu et al. [91] and Jiang et al. [92], substituting the hardware circuit with a neuromorphic transistor, significantly increased the capabilities of the wearable device. According to this approach, sensors and neuromorphic devices design can become more free, but require more energy consumption and are less wearable. The use of organic material in the preparation of neuromorphic transistors has a better compatibility with near-sensor perception systems compared to hardware circuits (Figure 2c–e). Fang et al. [93] introduced a new perception system with full flexibility, an integrated flexible pressure sensor and a flexible VO_2_ insulator-metal transition memristor on a polyethylene naphtholate two formic acid glycol ester (PEN) substrate (Figure 2f). This system needs a minimum pressure input of 3.82 kPa and a frequency output of more than 9.6 kHz. The structure of a capacitive sensor is like that of the piezoresistive sensors. The difference between them is the capacitance and resistance, respectively. What this means is that the functional layer can be prepared using non-conductive materials. Kim et al. [94] designed a capacitive sensor with PDMS + Ag flakes + HA, MIBK or Chloroform sinter-free ink as the electrodes and PDMS as the dielectric layer. The sensor can respond to both pressure and stretching (Figure 2g). It shows an excellent strain-insensitive performance, with only 0.002 of sensitivity per 1% strain at 120% stretching and a pressure sensitivity of 0.64 kPa^−1^ ranging from 0 to 1.8 kPa. A flexible Al/TiO_2_/Al memristor was used to construct the near-sensor perception system along with a flexible sensor. The weights trained by the neural network were mapped to the conductance of the memristor array, allowing the system to provide feedback on a specific pressure pattern input.

Piezoelectric and triboelectric sensors can operate without a power supply in contrast to piezoresistive and capacitive sensors [95]. A ceramic material is useful due to the strong piezoelectric effect [96,97]. Jung et al. [98] created a flexible artificial mechanoreceptor inspired by human skin. The HZO/TaN memristor and SiO_2_ electric insulation layer as the memory and HZO w/Al_2_O_3_ as the sensor were deposited onto a MICA film layer by plasma-enhanced chemical vapor deposition (Figure 2h). This integration of a sensor and front-end processing units onto a single flexible substrate can decrease the size of the perception system and increase its practicality. The sensor exhibits excellent performance in linearity within the range of 2~25 kPa. This system uses a non-volatile memristor as the front-end processing to demonstrate Braille recognition with an accuracy of up to 90.8% which was verified by deep neural network (DNN) software. Similarly, a triboelectric sensor without a power source can electrify through friction. Han et al. [99] designed a triboelectric nanogenerator (TENG) with an Al/PTFE/Al sandwich structure including a biristor neuron (Figure 2i). When pressure in the range of 3.3~5.3 kPa is applied, a small shift between the Al electrode and polytetrafluoroethylene (PTFE) can produce charges of opposite polarity and current in the circuit. The biristor neuron can output spikes of different frequency with the current generated by TENG. As a result, the TENG–biristor neuron can convert pressure signals into frequency signals, like the tactile receptors in human skin. For MNIST dataset deducing, SNN utility in the back-end unit can achieve an accuracy of 85.8%. Piezoelectric and triboelectric sensors also have a great potential in edge computing due to their self-powering abilities. This system works properly within the circumstances of continuously changing pressure.

In conclusion, the feasibility of a near-sensor tactile system has been verified by a number of studies. Compared to a conventional sensor–ADC–backend system structure, the consumption of the near-sensor tactile system reduces 1~2 orders of magnitude due to the redundant data decline [23]. However, a simpler system with one sensor and one neuromorphic device still faces a clear limit in terms of detecting and recognizing pressure patterns. Therefore, it is necessary to design and construct a stable and uniform flexible near-sensor tactile system array for wearable devices.

### 3.2. Flexible Near-Sensor Thalposis System

**Figure 3:**
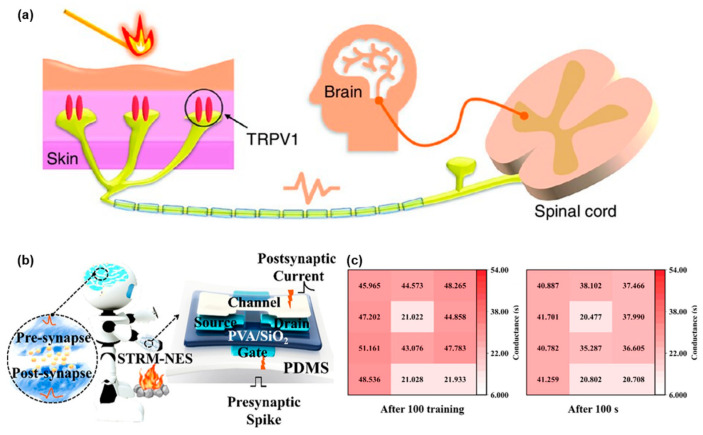
Near-sensor thalposis system. (**a**) Neural pathway of thalposis system in human beings. Reprinted with permission from Ref. [101]; Copyright 2022 John Wiley and Sons. (**b**) Diagram of stretchable temperature-responsive multimodal neuromorphic electronic skin (STRM-NES). (**c**) Conductance of the device after training with a letter “P” shaped heat source and its conductance after 100 s. Reprinted with permission from Ref. [108]; Copyright 2022 American Chemical Society.

The thalposis system is the part of the somatosensory system which allows humans to avoid frostbite and burns (Figure 3a). In contrast to the tactile system, which is used for object surface topography recognition and engages in daily communication, the temperature perception system only allows humans to sense temperature magnitude. Temperature can be measured by calculating the voltage at two ends of a thermistor or the current through it [100,101]. However, researching the neuromorphic temperature perception system is meaningful for electronic skin design for humanoid robots. A temperature sensor can be roughly categorized as either a thermocouple or a thermistor sensor. A thermocouple sensor calculates temperature by detecting the potential difference between two ends of a metal wire, while a thermistor sensor measures temperature according to changes in the thermistor resistance layer. Both thermocouple sensors and thermistor sensors are used in the sensing units of near-sensor thalposis systems with their own advantages, respectively. Compared to a thermocouple sensor, a thermistor sensor has a higher accuracy and a smaller size, making it capable of detecting slight temperature changes accurately and integrating easily. However, their linearity is not good. Flexible temperature sensors are typically prepared using carbon series materials (such as CNTs, rGO and graphite) and semiconductor films with high temperature coefficients (such as MoS_2_, MCNO and Ag_2_S) due to their compatibility with flexible organic polymers [102,103,104,105,106,107]. Thermistor sensors are often used in near-sensor systems because their thermal resistance mechanism and the electrical signal input requirements of their front-end processing units are well studied. Wang et al. [108] introduced a self-healing multimodal electronic skin for deformation and temperature perception. This system has a three-terminal transistor structure with a carbon nanotube (CNT) channel and CNT electrodes. The semi-conducted part and the metallic part are isolated by PVA/SiO_2_ (Figure 3b) and its temperature detection ranges from 20 °C to 50 °C. Similar to living skin, this system can break down in an environment with a temperature higher than 50 °C. Yet the dispersed PVA chain will cross-link; in other words, it can return back to its normal condition after the temperature returns to below 50 °C. Different temperatures can result in varying drain currents from the transistor. The temperature of the ambient environment affects the conductivity of the transistor array, and the distribution of temperatures can be directly reflected by the distribution of conductivity (Figure 3c).

Current research on near-sensor thalposis systems is still in its primary stages. They are only used to help increase the accuracy of pressure pattern recognition in tactile receptors. However, a high-performance pressure sensor array with a neural network would be better than bioreceptors. Therefore, research into temperature pattern recognition is no longer an urgent priority. However, the near/in-sensor architecture and neuromorphic device offer more unique advantages in classification and recognition. Therefore, thalposis system research should mainly concentrate on preparing large-scale arrays.

### 3.3. Flexible Near-Sensor Visual System

**Figure 4:**
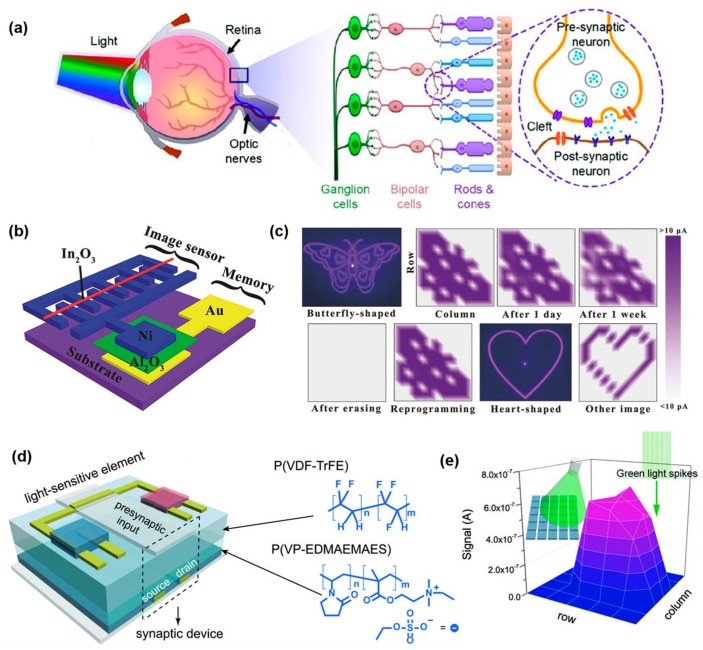
Near-sensor visual system. (**a**) Neural pathway of visual system in human beings. Reprinted with permission from Ref. [109]; Copyright 2020 John Wiley and Sons. (**b**) Structural diagram of the image sensor integrated with memristor as front-end processing unit. (**c**) Devices after modulated by butterfly-shaped and heart-shaped ultra-violet light source and its PSC accordingly. Reprinted with permission from Ref. [121]; Copyright 2018 John Wiley and Sons. (**d**) Structural diagram of the light-triggered organic neuromorphic device (LOND). (**e**) Devices triggered by green light spikes and display the illumination region through PSC. Reprinted with permission from Ref. [122]; Copyright 2018 John Wiley and Sons.

The visual system allows humans to locate objects and recognize visual information. It is composed of photoreceptors, a visual pathway and a visual center (Figure 4a) [109]. Capturing visual information (i.e., optical signals) and converting neural signal photoreceptors helps the visual center to make judgements and respond in time. Additionally, the visual system can enhance the recognition accuracy of other perception systems. A near-sensor visual system inspired by the human visual system would have the characteristics of a low power consumption and a robust information processing ability [110,111,112]. Thanks to the present technology, a flexible light-sensitive unit for artificial visual systems can be prepared easily. However, designing a near/in-sensor visual system is complex because of the requirements of capturing moving objects and accurately recognizing images. Therefore, the near/in-sensor visual system design focuses on the front-end processing units and back-end units.

The working mechanism of a photosensor is based on the photoelectric effect or photoconductivity of the semiconductor material. Valency electrons in PN junctions absorb photons and transit them into the conduction band with light illumination. This will produce free electrons and holes. In this way, an optical signal can be converted into an electrical signal. Semiconductor materials such as silicon, indium oxide, and carbon series materials combined with flexible organic polymers are used to prepare flexible photosensors [113,114,115,116,117,118]. To improve flexibility, rigid materials are shaped into nano size, such as nanotubes, nanorods and nanowires [119,120]. A strong information processing capability is required for both the front-end units and back-end units. Here, both neuromorphic devices and convolution neural networks (CNNs) are used to recognize optical images input from photosensors accurately. Chen et al. [121] designed a flexible printing platform integrated with a Ni/In_2_O_3_ semiconductor micrometer-size wires (SMWs)/Ni photosensor and a Ni/Al_2_O_3_/Au nonvolatile memristor on it (Figure 4b). The resistance of the photosensor can switch between a high resistance state and low resistance state under modulating UV light as low as 0.528 mW/cm^2^. As the photosensor and memristor are connected in parallel, a large enough voltage in the memristor can switch it from the OFF state to the ON state and maintain its state after turning off the power. Furthermore, a large-scale array was developed to identify butterfly and heart-shaped patterns. Inputs of different shapes can be easily distinguished by measuring the current output from the memristor (Figure 4c). Wang et al. [122] integrated a fully organic flexible visual system with an organic heterojunction as a photosensor and an organic synaptic transistor. This system can detect incident light at a wavelength of 850 nm and wavelength of 0.08 mW/cm^2^. The intensity and irradiated area of the incident light can be visualized by a simple readout of the post-synaptic current (PSC) (Figure 4d,e).

Research on artificial visual systems has focused on the in-sensor system benefits of novel materials discovered to have both light-sensitive and brain-like properties. The in-sensor visual system has a smaller size but larger scale integration capability compared to the near-sensor visual system. Section 4.2 will provide a detailed review of the in-sensor visual system.

### 3.4. Flexible Near-Sensor Olfactory System

**Figure 5:**
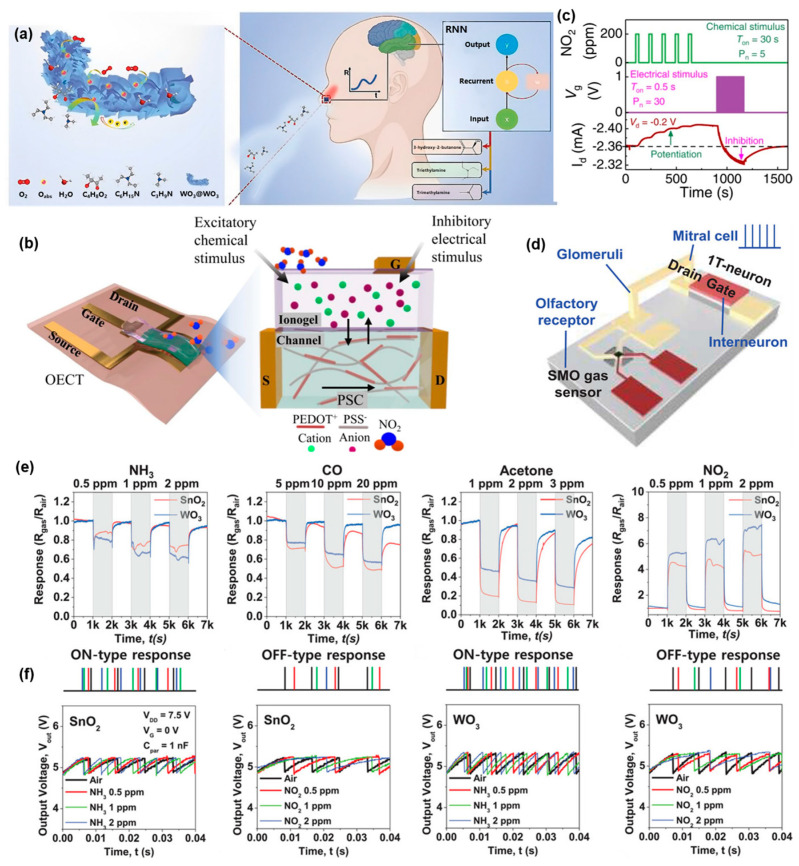
Near-sensor olfactory system. (**a**) Neural pathway of olfactory system in human beings. Reprinted with permission from Ref. [127]; Copyright 2024 John Wiley and Sons. (**b**) Structural diagram of the NO_2_ sensitive organic electrochemical transistor (OECT). (**c**) Electrical characteristics of OECT modulated by 200 ppm NO_2_ concentration and gate voltage. Reprinted with permission from Ref. [128]; Copyright 2023 Springer Nature. (**d**) Structural diagram of the SMO gas sensor integrated with transistor neuron. (**e**) Current response of SnO_2_ and WO_3_ gas sensor under same gas stimuli. (**f**) Frequency response of the device under same gas stimuli. Reprinted with permission from Ref. [129]; Copyright 2022 John Wiley and Sons.

The olfactory system helps us to cognize specific objects from their unique scent. Essentially, this system needs to distinguish various molecules and respond accordingly for a proper working condition [123,124]. An olfactory sensor which is inspired by the human olfactory system and based on chemical sensing principle can detect the concentration and type of various molecules through chemically reactions (Figure 5a). To some extent, the design of a flexible sensor depends on the molecules to be detected. For instance, a PDMS/Ppy double layer nanostructure, which is used to detect organic molecules through its triboelectric/gas-sensing coupling effect, can detect acetone concentrations as low as 100 ppm [125]. Similarly, single-walled carbon nanotubes (SWCNTs) combined with olfactory receptor-derived peptides (ORPs) are useful for detecting harmful concentrations of trimethylamine (TMA) with a lower limit of 0.01 ppq [126]. Through the chemical reaction, chemical molecules can transform information into electric signal [127]. Instead of inorganic material, organic material is more prevalent in olfactory sensors due to their greater variety of chemical bonds. Chouhdry et al. [128] designed an organic electrochemical transistor (OECT) with a PEDOT:PSS channel, a poly(ethylene glycol) diacrylate (PEGDA) monomer and a 1-hydroxycyclohexyl phenyl ketone photo initiator as the chemoreceptive material, as well as a gate electrolyte layer. An ion gel made of [EMIM][TSFI] was produced as a gas sensing layer, connected to the gate of the OCET (Figure 5b). This near-sensor olfactory system can detect NO_2_ concentrations starting from 2.66 ppm. Its output exhibits long-term potentiation and depression effects (Figure 5c). Han et al. [129] designed a type of semiconductor metal oxide gas sensor with SnO_3_ and WO_3_, respectively, and a neuron integrated into it with traditional metal oxide semiconductor field effect transistor (MOSFET) chips (Figure 5d). Gas sensors can capture specific gas concentrations with a lower limit of 0.5 ppm via resistance changing and a loop current (Figure 5e). This loop current determines the output frequency from the 1T-neuron. The output of gas sensors made from SnO_3_ or WO_3_ can be another input of a neural network for gas recognition and classification (Figure 5f).

The combination of a near-sensor olfactory system and a flexible device as a more intelligent molecular detector has occupied a key seat in current research on harmful gases detection. Flexible gas sensors have a ultra-high quality of detection sensitivity compared to the biological olfactory system, particularly a lower measurable threshold. However, due to the material utility of a gas sensor, only a few gas molecules can be detected through chemical reactions in the sensitive layer. Thus, expanding the range of detectable molecules and designing the back-end unit for molecule classes distinction are still challenges we are currently facing.

### 3.5. Flexible Near-Sensor Auditory System

**Figure 6:**
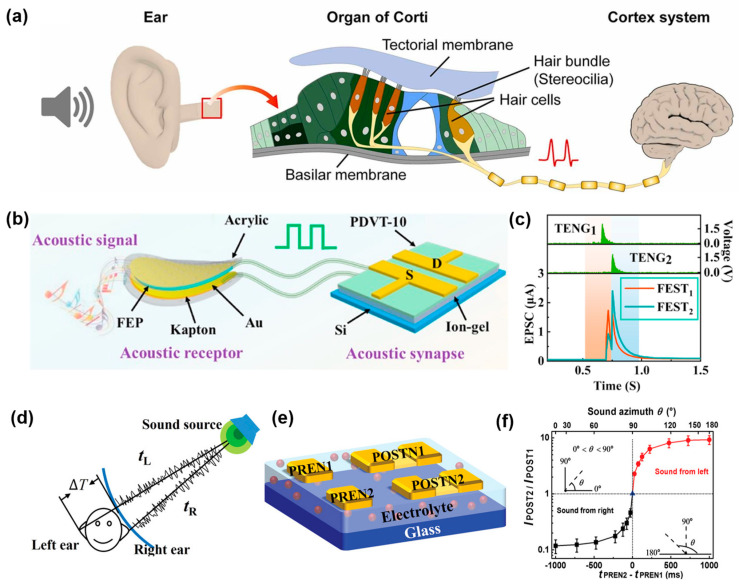
Near-sensor auditory system. (**a**) Neural pathway of olfactory system in human beings. Reprinted with permission from Ref. [135]; Copyright 2023 Elsevier. (**b**) Structural diagram of the acoustic sensor connected with synaptic transistor. (**c**) PSC current of FEST_1_ and FEST_2_ when the sound comes from the right direction. Reprinted with permission from Ref. [139]; Copyright 2020 Elsevier. (**d**) Schematic image of the binaural effect. (**e**) Structural diagram of dual-gate synaptic transistor. (**f**) Function of the sound source azimuth estimation by calculating the ratio of the PSC of two transistors. Reprinted with permission from Ref. [140]; Copyright 2019 John Wiley and Sons.

The biological auditory system enables humans and other species to locate the source of sound and identify meaningful information (Figure 6a). Similarly, the artificial auditory system can locate a sound source through binaural effects and identify acoustic information by perceiving frequency and amplitude [130,131,132]. Indeed, the auditory sensor converts frequency and amplitude signals into electrical signals for the front-end processing units. For flexible auditory sensors in a near-sensor auditory system, a low detection limit is necessary to detect the small mechanical vibrations caused by sound waves [133,134]. An electromagnetic material is a typical component of an auditory sensor with a high sensitivity and fast response. Additionally, high-sensitivity pressure sensor can be used as auditory sensors due to their ability to detect of micro vibrations [135,136,137,138]. Liu et al. [139] designed a triboelectric nanogenerator (TENG) with an Au/fluorinated ethylene propylene (FEP) film/Kapton film for acoustic signal detection. Furthermore, they developed a synaptic transistor with a PVDT-10 channel and an [LI][TSFI] ion gel dielectric layer (Figure 6b) to capture and accumulate waves in the frequency range of 50–5000 Hz. The TENG can output a voltage of 126 mV/dB. Two TENGs and two field effect synaptic transistors (FESTs) are used to locate and determine the orientation of a sound source by calculating the amplitude ratio of two FESTs (Figure 6c). The FEST can also accurately detect and filter acoustic signals in noisy environments during processing. He et al. [140] designed a multi-gate synaptic transistor with an IGZO channel and a chitosan solid electrolyte film. This transistor can simulate the binaural effects of the human auditory system (Figure 6d,e). Like the disparate position of a human’s two ears, auditory sensor will receive the signals with different strengths according to their position. The distance between the sensors and the sound source can be measured by measuring the postsynaptic current (PSC) produced from audio signals. The approximate position of the sound source can be obtained by calculating the amplitude ratio of the PSC in a synaptic transistor (Figure 6f).

Differently to other systems, research on the artificial auditory system is relatively complete, including as the creation of a flexible device, hardware system and circuit match. Thanks to significant advances in artificial intelligence, the characteristics of acoustic signals extracted by neuromorphic devices can be accurately recognized by a neural network. A higher compatibility of all of the parts in a near/in-sensor system is needed for a wearable intelligent system which is integrated with a perception system.

## 4. Flexible In-Sensor Artificial System

On the basis of a near-sensor artificial system, the front-end processing unit and sensing unit need to be integrated into in-sensor systems further and entirely. In-sensor computing architecture is smaller and consumes less power than near-sensor computing architecture [141,142]. Sensors that combine sensing, storing and computing functions or sensing and computing functions are referred to as multi-functional sensing units. The in-sensor computing architecture eliminates the redundant data between the sensing and computing units by data pre-processing in the former part [21]. Implementing powerful features into in-sensor artificial systems, such as material selecting and device structure designing, is still a challenge in this field despite their considerable advantages. Replacing electro-resistive materials with other sensing signal-induced resistive materials is a relatively good design solution for the introduction of a novel material to neuromorphic devices. Combining sensors and neuromorphic devices into a unified entity as a new integrated structure is another solution.

### 4.1. Flexible In-Sensor Tactile System

**Figure 7:**
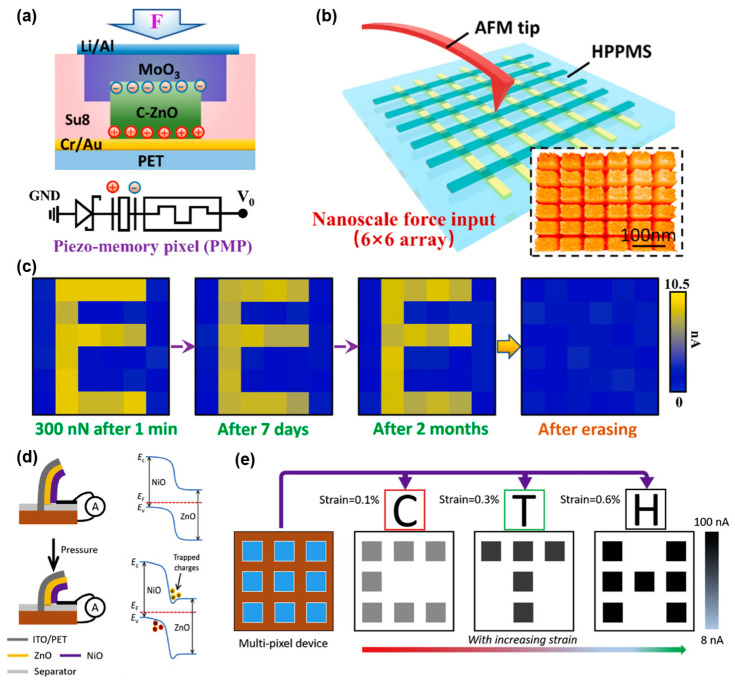
In-sensor tactile system. (**a**) Schematic image of the PMP. (**b**) AFM image and diagram of the 6 × 6 array. (**c**) Pressure signal perception and storage characteristic. Reprinted with permission from Ref. [146]; Copyright 2021 Elsevier. (**d**) Schematic image of the in-sensor system inspired by the fine hair on the human body. (**e**) Response of letter “C”, “T”, “H”-shaped pressure inputs under different strain. Reprinted with permission from Ref. [147]; Copyright 2020 Elsevier.

For an in-sensor tactile system, materials with both stress-response and synaptic characteristics have not yet been proposed. Therefore, integrating pressure-sensitive materials and resistive materials into a single device and designing a rational structure has become the main course of action [143,144]. Jiang et al. [145] designed a flexible multi-functional sensing unit for a complex structure with a PET substrate, Cr/Au bottom electrode, Su8/C-ZnO/Su8 pressure sensitive layer, MoO_3_ resistive layer and Li/Al upper electrode (Figure 7a). In addition, ZnO nanowire is a common piezoelectric material used for self-power supply instantiation. It can generate a voltage of 0–5 V under a pressure of 0–400 nN. A 6 × 6 piezo memory pixel (PMP) array (Figure 7b) is used to store English letters with considerable durability (Figure 7c). What is more, Kumar et al. [146] designed a PET/ITO/ZnO/NiO flexible film inspired by the mechanoreceptor in human skin. Because of the difference in electron affinity between ZnO and NiO, charges can be trapped in NiO under the pressure, which is similar to the P-N junction. Through this mechanism, the conductance of the device can be modulated by applied pressure (Figure 7d). Specifically, the same number of spikes can generate different currents with different pressures. Additionally, a 3 × 3 array can store English letters. The PSC of the device can be modulated by pressure and strain (Figure 7e). To improve the performance of in-sensor tactile system, a new mechanism for easily preparing materials and integrating multi-functional sensing units is necessary.

### 4.2. Flexible In-Sensor Visual System

**Figure 8:**
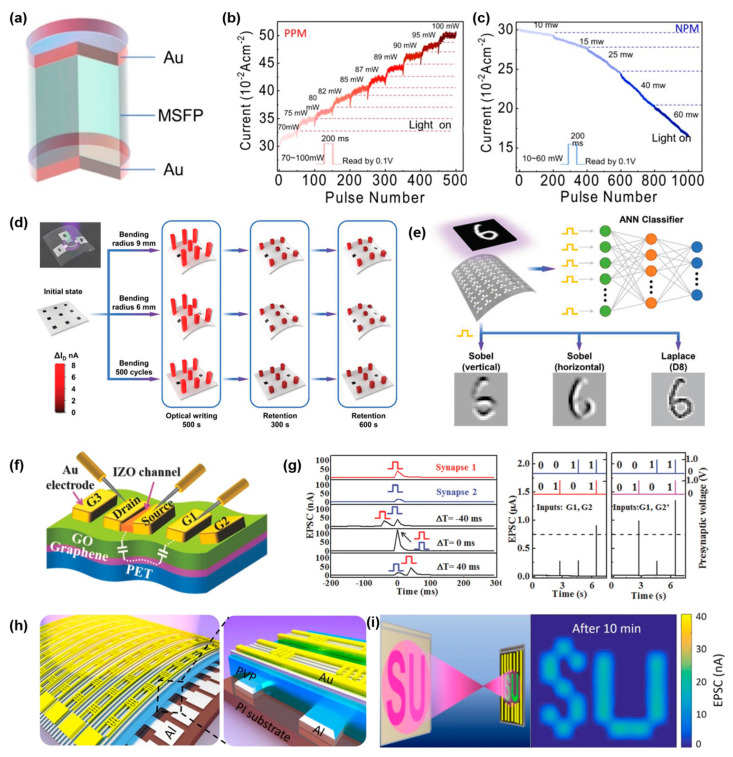
In-sensor visual system. (**a**) Structural diagram of the Au/MSFP/Au light sensor. (**b**,**c**) Optical modulation characteristics of the device; positive and negative effects are bounded by 60 mW. Reprinted with permission from Ref. [153]; Copyright 2023 Springer Nature. (**d**) Data storage capability of the 3 × 3 array with good bending stability. (**e**) ANN is used as the back end to classify MNIST dataset. Reprinted with permission from Ref. [154]; Copyright 2022 John Wiley and Sons. (**f**) Structural diagram of the multi-gate transistor. (**g**) Implementation of AND/OR logic function by multi-gate modulation. Reprinted with permission from Ref. [155]; Copyright 2016 John Wiley and Sons. (**h**) Structural diagram of a large-scale photo synaptic device array. (**i**) Storage capability of the photo synaptic device array. Reprinted with permission from Ref. [156]; Copyright 2019 Springer Nature.

Compared to the near-sensor visual system, the in-sensor visual system has been systematically researched due to the proposal of a wide variety of photo-induced resistive materials [147,148,149,150,151,152]. This means that neuromorphic devices can be modulated by an optical spike being directly dropped into the procedure of electric spike converting. Integrating a single multi-function sensing unit with two functional units (a sensor and a transistor) is beneficial for a further large-scale integration. Zhou et al. [153] designed an Au/modified silk fibroin protein (MSFP)/Au resistive random-access memory (RRAM) with a simple sandwich structure (Figure 8a). The resistance of the device can be transferred from HRS to LRS with a light intensity with a power of greater than 60 mW, and can be transferred to HRS from LRS with a power less than 60 mW (Figure 8b,c). Hardware contrast enhancement and background denoising can be implemented via these features. Li et al. [154] designed a flexible VO_2_/mica film with Pt electrodes for crossbar array preparation. This VO_2_-based light-modulated memristor exhibits good bending stability and a long retention time for the conductance state (Figure 8d). Furthermore, this device can be modulated by visible light with an intensity of greater than 1 mW/cm^2^. Combining the memristor array and artificial neural network (ANN) classifier demonstrates the system’s powerful intelligence in MNIST dataset recognition and motion detection (Figure 8e). Wan et al. [155] designed a multi-gate flexible neuromorphic transistor with a PET/graphene/GO/Au layered structure (Figure 8f). A device with an IZO film as a channel can output different PSCs based on the distance between the channel and gate. By setting reasonable thresholds or changing the position of the gate, different basic logic functions such as AND, OR or XOR can be realized (Figure 8g). Deng et al. [156] designed a Dif-TES-ADT crystal material with the light response characteristic. It was combined with a PVP dielectric layer, Al gate and a Au electrode for manufacture of photo synaptic device (Figure 8h). This device can accumulate light spikes with an incident light intensity ranging from 0.001 to 53μW/cm^2^. Additionally, they prepared a large-scale array for long-term storage of input optical images (Figure 8i).

Currently, flexible photo-induced resistive materials and circuit designs for in-sensor visual systems have extended, resulting in significant progress. However, the biological visual system has more powerful and complex functions than the function-specific artificial visual system. Furthermore, integrating the visual system with other compatible artificial sensory systems is still a challenging next step.

## 5. Summary and Outlook

The near/in-sensor computing paradigm is an emerging solution for real-time and data-intensive applications. Processing data directly at the sensor terminal significantly improves the area, time and energy efficiency of hardware. Inspired by the brain, low-level processing such as filtering and denoising can be implemented by neuromorphic devices at the sensor terminal. For the IoT, the near/in-sensor computing architecture is the best choice for wearable application due to its real-time capability and low power consumption. The sensing units and front-end units in near-sensor architecture can be designed separately because of their physical separateness. Therefore, materials such as inorganic films and organic semiconductors can be used for flexible device fabrication. For in-sensor architecture, selecting the flexible material carefully is beneficial for entirely integrating the sensor and the neuromorphic device. Although near/in-sensor systems show great potential in practical applications, most devices are still in an early stage of development. Because of their limited functionality, current near/in-sensor systems can only be used in specific scenarios. Near/in-sensor computing involves multiple disciplines such as materials, circuits and algorithms. The back end is an important part in the design of wearable smart devices. To fully realize a near/in-sensor computing system, all parts of the system must be simultaneously optimized. From near-sensor architecture to in-sensor architecture, to implement high-level processing at the sensor terminal, continuous optimization of the material, device and circuit design is required. This will allow for autonomous monitoring and the analysis of physiological signals without relying on cloud-based resources and will also make implementing wearable devices with a lower power consumption and greater intelligence possible.

From the perspective of developing high performance wearable devices, replacing traditional computing architecture with low-power near/in-sensor computing architecture is extremely possible. The emerging neuromorphic devices have the possibility of realizing near/in-sensor perception systems, but it is hard to achieve an operational stability equivalent to that of MOSFETs. Therefore, devices with a ferroelectric mechanism or three terminals are often used to improve the stability of the system. The design of an in-sensor system is more difficult and complex than that of an near-sensor system. What this means is that more ground-breaking designs with novel structure and mechanism of device are required. Meanwhile, the additional requirement of the design of flexible material is also a problem that needs solving. Because of the advantages of portability and low energy consumption, from the perspective of application and the market, wearable devices with a flexible near/in-sensor system are likely to squeeze the market space of traditional artificial intelligence devices in future market competitions. Due to their portability and user experience, traditional wearable devices have gained a significant market share from rigid device. However, the increasing functionality of these devices means that it takes a higher power consumption to impair the battery life. Furthermore, more generated data need to be uploaded for cloud processing. Traditional wearable devices meet more difficulty in current market environment with these increasing drawbacks. Creating flexible near/in-sensor systems, with an in situ information processing ability, further reduces the power consumption and data transfer latency. These advantages help them to hold the trophy in the wearable device market and make them eligible to compete with AI-based devices. Although traditional architecture combined with ANN is popular in current wearable smart devices, we strongly believe that the next generation of wearable intelligent devices will greatly benefit from the integration of near/in-sensor computing architecture and neuromorphic devices.

## Figures and Tables

**Table 1:** Key parameters and functions of different systems.

System Type	Key Parameter	Application
Tactile	Sensitivity, detection range	Pattern recognition
Thalposis	Detection range	Temperature perception, Assistance
Visual	Detection limit, conversion rate	Image recognition, assistance
Olfactory	Detection limit, molecular species	Molecular distinguishing
Auditory	Detection limit, frequency range	Location, audio recognition

## Abbreviations

IoT	Internet of Things
ADC	Analog-to-Digital Converter
SFL	Sensor Function Layer
DNN	Deep Neural Network
TENG	Triboelectric Nanogenerator
CNN	Convolutional Neural Network
PSC	Postsynaptic Current
ANN	Artificial Neural Network
MOSFET	Metal Oxide Semiconductor Field Effect Transistor
